# Supplementary material for: Does controlling shareholders’ share pledge exacerbate excessive financialization of enterprises?—Evidence from performance pressure perspective
Source: PLoS One. 2023 Jul 19;18(7):e0288705. doi: 10.1371/journal.pone.0288705 (PMC10355460; doi:10.1371/journal.pone.0288705)
Supplement: Attachment — Submitted filename: Response to Reviewers.docx [file pone.0288705.s003.docx]

Authors’ Response to the Reviewers’ Comments

PONE-D-23-11287

“Does Controlling Shareholders' Share Pledge Exacerbate Excessive Financialization of Enter-prises? ——Evidence from Performance Pressure Perspective”

by

Yue Xie, Tianhui Wang, Jinhua Zhang and Na Wang

We would like to thank the referees and the editor for their helpful comments and constructive suggestions. We have revised the paper accordingly, the major changes are marked in blue. In the following, we give our responses to the reviewer. And we mention the changes made in the revised manuscript. All of the major changes are marked in blue in the revised version.

**Response to the Academic Editor:**

**Editor’s additional comments:**

1. After careful consideration, we feel that it has merit but does not fully meet PLOS ONE’s publication criteria as it currently stands. Therefore, we invite you to submit a revised version of the manuscript that addresses the points raised during the review process.

**Authors’ Response: The authors would like to thank the Editor for the comment. We have addressed the comments of the Reviewer. In the following, we give our responses to the comments of the Reviewer.**

2. The authors need to link their findings more strongly to context, highlight their economic, academic/research and policy/practice implications.

I may suggest the authors add the discussion of the findings in the Chinese context. How their findings are different from other studies? What can we learn from the Chinese context?

**Authors’ Response: Thanks for the comment. We have added the research significance in the introduction in the revised manuscript. At the same time, we have revised the expression of contribution at the end of the introduction and the expression of the conclusion in the revised manuscript.**

**Response to the Editor:**

**Journal Requirements:**

1. Please ensure that your manuscript meets PLOS ONE's style requirements, including those for file naming.

**Authors’ Response:** **Thanks for reminding me. I have modified the paper format according to the journal template. If the format needs to be modified, please inform me again by email. Thank you.**

2. We note that the grant information you provided in the ‘Funding Information’ and ‘Financial Disclosure’ sections do not match.

When you resubmit, please ensure that you provide the correct grant numbers for the awards you received for your study in the ‘Funding Information’ section.

**Authors’ Response: Thanks for reminding me. The correct information is as follows:**

**Funding:** This research was funded by the National Natural Science Foundation of China (grant number: 72002201), URL of funder website: <https://www.nsfc.gov.cn/>; And the National Social Science Fund of China (grant number: 21BJY256), URL of funder website: <http://fz.people.com.cn/skygb/sk/index.php/index/seach/>

3. Please review your reference list to ensure that it is complete and correct. If you have cited papers that have been retracted, please include the rationale for doing so in the manuscript text, or remove these references and replace them with relevant current references. Any changes to the reference list should be mentioned in the rebuttal letter that accompanies your revised manuscript. If you need to cite a retracted article, indicate the article’s retracted status in the References list and also include a citation and full reference for the retraction notice.

**Authors’ Response: Thanks for reminding me. After careful examination, the references we quoted are complete and correct. We did not cite the retracted paper. In the contribution at the end of the introduction, we add the literature that is more similar to our study, namely 5-7 in the references. Except for the three new references, we have not changed the rest of the references.**

**Response to the Reviewer #1**

The reviewer has the following comments:

1. The authors need to link their findings more strongly to context, highlight their economic, academic/research and policy/practice implications.

**Authors’ Response: Thanks for the comment. We have added the research significance in the introduction in the revised manuscript.**

2. I may suggest the authors add the discussion of the findings in the Chinese context.

**Authors’ Response:** **Thanks for this suggestion. We have revised the expression of the conclusion in the revised manuscript.**

3. How their findings are different from other studies? What can we learn from the Chinese context?

**Authors’ Response:** **Thanks for this suggestion. We have revised the expression of contribution at the end of the introduction in the revised manuscript.**

**Response to the Reviewer #2**

The reviewer has the following comments:

1. An interesting topic, check for grammar and conjugation errors and add the article at the end of the introduction.

The database is recent and the analyses are very impressive.

I congratulate the authors for this article.

**Authors’ Response:** **Thank you for your suggestions and affirmation of our paper. We have revised the grammar and conjugation errors in the revised manuscript. At the same time, we have added some articles similar to our paper at the end of the introduction in the revised manuscript.**

**We tried our best to improve the manuscript and made some changes in the manuscript. These changes will not influence the content and framework of the paper. And here we did not list all the changes but marked in blue in revised paper.**

**We appreciate for Editors/Reviewers’ warm work earnestly, and hope that the correction will meet with approval.**

**Once again, thank you very much for your comments and suggestions.**
